# Testing for *ROS1* in non-small cell lung cancer: a review with recommendations

**DOI:** 10.1007/s00428-016-2000-3

**Published:** 2016-08-17

**Authors:** Lukas Bubendorf, Reinhard Büttner, Fouad Al-Dayel, Manfred Dietel, Göran Elmberger, Keith Kerr, Fernando López-Ríos, Antonio Marchetti, Büge Öz, Patrick Pauwels, Frédérique Penault-Llorca, Giulio Rossi, Aleš Ryška, Erik Thunnissen

**Affiliations:** 1 Institute of Pathology, University Hospital Basel, Basel, Switzerland; 2 Institute of Pathology, University Hospital Cologne and Network Genomic Medicine, Cologne, Germany; 3 Department of Pathology and Laboratory Medicine, King Faisal Specialist Hospital and Research Centre, Riyadh, Saudi Arabia; 4 Institute of Pathology, Charité Campus Mitte, Berlin, Germany; 5 Department of Pathology and Cytology, Karolinska University Hospital, Stockholm, Sweden; 6 Department of Pathology, Aberdeen University Medical School, Aberdeen, UK; 7 Laboratorio de Dianas Terapéuticas, Hospital Universitario HM Sanchinarro, C/Oña, 10, 28050 Madrid, Spain; 8 Center of Predictive Molecular Medicine, University-Foundation, Chieti, Italy; 9 Cerrahpasa Medical Faculty, Istanbul University, Istanbul, Turkey; 10 Institute of Pathology, University Hospital Antwerp, Edegem, Belgium; 11 Department of Pathology, Centre Jean Perrin, Clermont-Ferrand, France; 12 Unit of Pathologic Anatomy, Azienda USL Valle dʹAosta, Aosta, Italy; 13 The Fingerland Department of Pathology, Charles University Faculty of Medicine and Faculty Hospital in Hradec Kralove, Hradec Kralove, Czech Republic; 14 Department of Pathology, VU University Medical Centre, Amsterdam, The Netherlands

**Keywords:** Fluorescence in situ hybridisation, Immunohistochemistry, Non-small cell lung cancer, Predictive marker, ROS1, RT-PCR

## Abstract

Rearrangements of the *ROS1* gene occur in 1–2 % of non-small cell lung cancers (NSCLCs). Crizotinib, a highly effective inhibitor of ROS1 kinase activity, is now FDA-approved for the treatment of patients with advanced *ROS1*-positive NSCLC. Consequently, focus on *ROS1* testing is growing. Most laboratories currently rely on fluorescence in situ hybridisation (FISH) assays using a dual-colour break-apart probe to detect *ROS1* rearrangements. Given the rarity of these rearrangements in NSCLC, detection of elevated ROS1 protein levels by immunohistochemistry may provide cost-effective screening prior to confirmatory FISH testing. Non-in situ testing approaches also hold potential as stand-alone methods or complementary tests, including multiplex real-time PCR assays and next-generation sequencing (NGS) platforms which include commercial test kits covering a range of fusion genes. In order to ensure high-quality biomarker testing, appropriate tissue handling, adequate control materials and participation in external quality assessment programmes are essential, irrespective of the testing technique employed. *ROS1* testing is often only considered after negative tests for *EGFR* mutation and *ALK* gene rearrangement, based on the assumption that these oncogenic driver events tend to be exclusive. However, as the use of ROS1 inhibitors becomes routine, accurate and timely detection of *ROS1* gene rearrangements will be critical for the optimal treatment of patients with NSCLC. As NGS techniques are introduced into routine diagnostic practice, *ROS1* fusion gene testing will be provided as part of the initial testing package.

## Introduction

Lung cancer is the most frequent cause of cancer-related death worldwide and is usually diagnosed in advanced stages [1]. The most common histological lung cancer subgroup is non-small cell lung cancer (NSCLC), which accounts for 80 % of lung cancers [1]. Currently, there are two identified molecular subtypes of NSCLC that have targeted therapies approved for their treatment: mutations in the epidermal growth factor receptor (*EGFR*) gene and rearrangements in the anaplastic lymphoma kinase (*ALK*) gene; tumours harbouring these genetic alterations respond well to specific tyrosine kinase inhibitors [2, 3]. In addition to *EGFR* and *ALK*, other known oncogenic drivers of NSCLC include hepatocyte growth factor receptor (*MET*), the GTPase *KRAS*, human epidermal growth factor receptor 2 (*HER2*), *RET* and *ROS1* [4, 5].

ROS1 is now recognised as a distinct molecular target in NSCLC [6, 7]. Pre-clinical and clinical studies demonstrate that ROS1 can be efficiently inhibited by the tyrosine kinase inhibitor crizotinib [8, 9], which is approved by the FDA and EMA as a treatment for patients with advanced *ALK*-positive NSCLC [10, 11]. Crizotinib was recently approved by the FDA for patients with advanced *ROS1*-positive NSCLC [12]; therefore, detection of *ROS1* gene rearrangements is critical for the optimal treatment of *ROS1*-positive NSCLC patients. In this article, we review the current state of molecular diagnostics for *ROS1*-positive NSCLC, discuss our experience with the relevant technologies and provide guidance on the detection of *ROS1*-positive tumours.

## Rationale for targeting ROS1 fusions in NSCLC

Although *v-ROS1* had already been identified as a unique oncogenic sequence in the avian sarcoma virus (VR2) [13], a chicken retrovirus, it was only in 2003 that the genomic structure of *ROS1* was fully characterised [14]. ROS1 belongs to the human receptor tyrosine kinase (RTK) family and is evolutionarily close to the ALK family, forming part of the scientific basis for using inhibitors of ALK as inhibitors of ROS1. The *ROS1* gene is located on chromosome 6 (6q22) and encodes a transmembrane receptor protein with unique features. The extracellular N-terminal domain spans more than 1800 amino acids, which makes it one of the largest extracellular domains amongst all human RTKs. Despite this, no human ROS1 ligand has been found to date and the physiological function of this orphan receptor is still unclear. The C-terminal portion of ROS1 contains a kinase domain and a single transmembrane domain [9, 15–17].

**Fig. 1:**
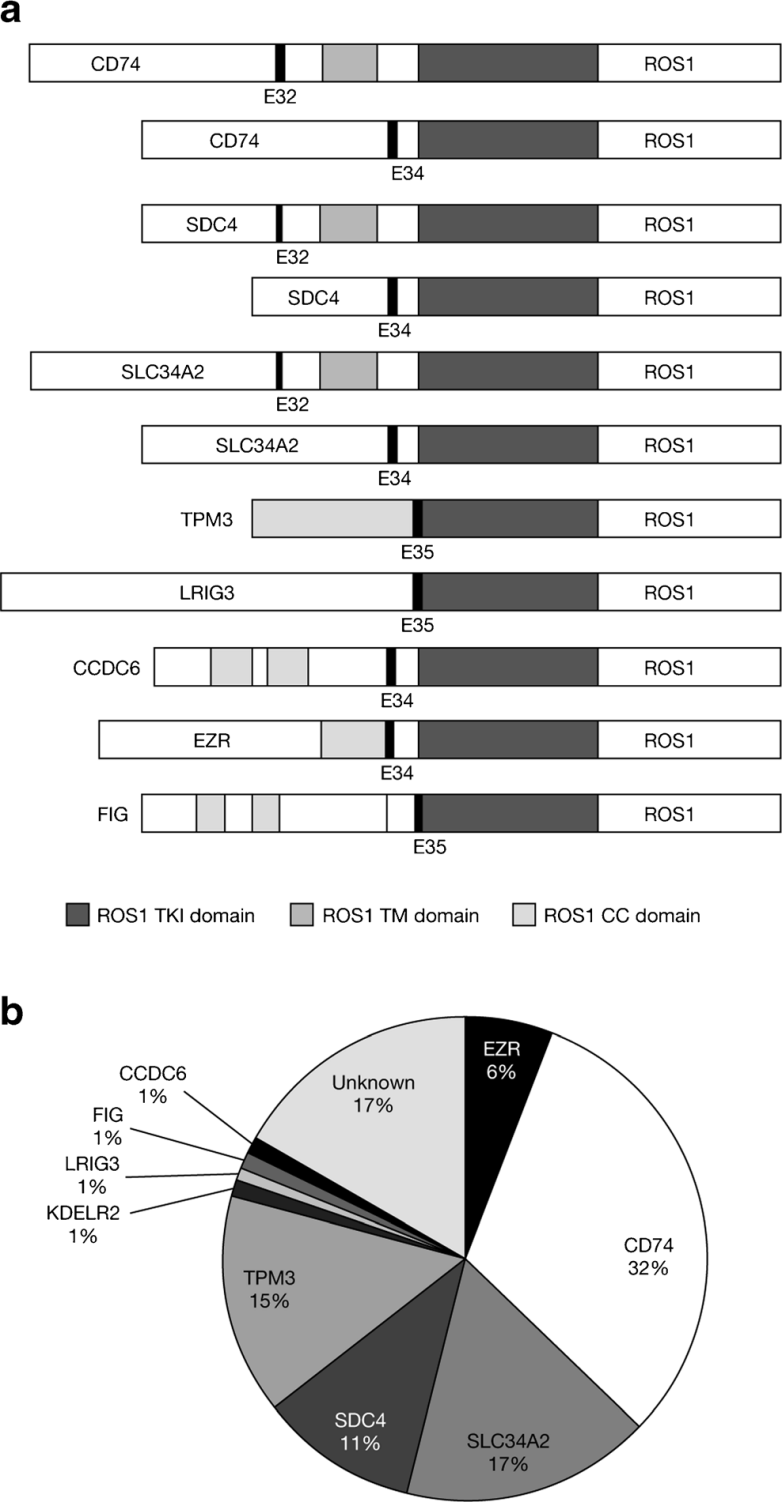
**a** Schematic diagram of *ROS1* fusions in NSCLC showing *ROS1* tyrosine kinase domain (TKI, *dark grey*), *ROS1* transmembrane domain (TM, *mid-grey*) and coiled-coil domains (CC, *light grey*) in *ROS1* fusion proteins (*KDELR2-ROS1* is not shown). Reproduced from Gainor and Shaw [35]. **b** Frequencies of different *ROS1* fusion partners. Adapted from Gainor and Shaw [35], with additional data from more recent studies as reported in Table 1

Genomic rearrangements involving *ROS1* occur in 1–2 % of NSCLCs [9, 18–23]. *ROS1* gene rearrangement was initially discovered in the glioblastoma cell line V118MG [24]. In this cell line, an intrachromosomal deletion on chromosome 6 fused the 5′ region of a gene named *FIG* to the 3′ region of *ROS1*. Since then, many more novel *ROS1* fusion partners have been found. Importantly, the ROS1 kinase domain is retained in all of these fusion events and the expressed fusion genes have been reported to be oncogenic. Known *ROS1* fusion partners in lung cancer include *FIG*, *CD74*, *SLC34A2* and *SDC4*, and the list is growing. *CD74-ROS1* is the most frequently detected *ROS1* fusion in this group of patients. With all of the known fusion genes, the ROS1 kinase domain is fully retained and the *ROS1* junction point at the messenger RNA (mRNA) level invariably occurs at the 5′ end of exons 32, 34, 35 or 36 (Fig. 1 and Table 1).

**Table 1:** Prevalence of *ROS1* rearrangements in non-small cell lung cancer screening studies (modified from Gainor and Shaw 2013 [35]^a^)

Study	Screening/validation techniques	Prevalence of ROS1 fusions	Rearrangements identified by fusion partner (no.)
Arai et al. [66]	Transcriptome sequencing, RT-PCR	4/569 (0.7 %)	(4) EZR
Bergethon et al. [9]	FISH, RT-PCR	18/1073 (1.7 %)	(5) CD74 (1) SLC34A2 (8) Unknown partner (4) Insufficient tissue
**Cai et al. [** 67 **]**	**RT-PCR, direct sequencing**	**8/392 (2.0 %)**	**(4) SLC34A2** **(3) CD74** **(1) SDC4**
**Cheng et al. [** 68 **]**	**FISH, Sanger sequencing**	**53/1652 (3.2 %)**	**(15) CD74** **(13) SLC34A2** **(13) SDC4** **(12) TPM3**
Davies et al. [69]	FISH, RT-PCR	5/428 (1.2 %) 1/48 (2.1 %)	(2) CD74 (2) SLC34A2 (1) SDC4 (1) SDC4
**Fu et al. [** 70 **]**	**FISH, direct sequencing, IHC**	**4/204 (2.0 %)**	**(3) SDC4** **(1) Negative on direct sequencing**
**Go et al. [** 71 **]**	**FISH, RT-PCR**	**16/515 (3.1 %)**	**(2) CD74** **(1) TPM3** (5) Tissue not available
Govindan et al. [18]	Whole-genome and transcriptome sequencing	1/17 (5.9 %)	(1) KDELR2
**Jin et al. [** 72 **]**	**FISH, IHC**	**3/375 (0.8 %)**	**Not reported**
**Karlsson et al. [** 73 **]**	**Massive parallel sequencing**	**0/73 (0 %)**	**No ROS1 fusions found**
**Kim et al. [** 74 **]**	**FISH, RT-PCR**	**7/208 (3.4 %)**	**(2) CD74** **(5) Unknown partner**
**Kirita et al. [** 75 **]**	**FISH, RT-PCR, IHC**	**2/70 (2.9 %)**	**Not reported**
Li et al. [76]	RT-PCR, direct sequencing	2/202 (1 %)^b^	(2) CD74
**Matsuura et al. [** 77 **]**	**RT-PCR, IHC**	**1/114 (0.9 %)**	**(1) CD74**
**Okamoto et al. [** 78 **]**	**RT-PCR, FISH**	**5/240 (2.1 %)**	**(3 SLC34A2)** **(1 LRIG3v1)** **(1 CD74)**
Rikova et al. [19]	Phosphoproteomics screen, RT-PCR	1/150 (0.7 %)	(1) CD74 (1) SLC34A2^c^
Rimkunas et al. [20]	IHC, RT-PCR, FISH	9/556 (1.6 %)	(4) CD74 (2) SLC34A2 (1) FIG (1) Unknown partner (1) Insufficient tissue
**Scheffler et al. [** 79 **]**	**FISH, NGS**	**19/1035 (1.8 %)**	**Not reported**
Seo et al. [21]	Whole-transcriptome sequencing, RT-PCR	3/200 (1.5 %)	(1) CD74 (1) SLC34A2 (1) CCDC6
Suehara et al. [80]	Messenger RNA screen, RT-PCR	1/69 (1.4 %)^d^	(1) FIG
Takeuchi et al. [22]	FISH, RT-PCR	13/1476 (0.9 %)	(3) CD74 (3) SDC4 (2) TPM3 (2) EZR (1) SLC34A2 (1) LRIG3 (1) Unknown partner
**Wang et al. [** 81 **]**	**RT-PCR**	**11/1356 (0.8 %)**	**Not reported**
**Warth et al. [** 82 **]**	**IHC, FISH**	**9/1478 (0.6 %)**	**Not reported**
Yoshida et al. [23]	RT-PCR, FISH	15/799 (1.9 %)	(10) CD74 (4) EZR (1) SLC34A2
**Zhang et al. [** 83 **]**	**FISH, IHC**	**2/120 (1.7 %)**	**Not reported**
**Zhao et al. [** 84 **]**	**RT-PCR, DNA sequencing**	**2/108 (1.9 %)**	**(2) TPM3**
**Zhong et al. [** 85 **]**	**RT-PCR, Sanger sequencing**	**12/302 (4.0 %)**	**(9) CD74** **(3) Not reported**

*FISH* fluorescence in situ hybridisation, *IHC* immunohistochemistry, *NGS* next-generation sequencing, *RT-PCR* reverse transcription polymerase chain reaction

^a^Entries shown in bold have been added to the table (other entries are as presented by Gainor and Shaw [35])

^b^Screened specimens consisted entirely of resected adenocarcinomas from never-smokers who were negative for alterations in *EGFR*, *KRAS*, *HER2*, *ALK* and *BRAF*

^c^Identified in cell line

^d^Screened specimens consisted of ‘pan-negative’ adenocarcinomas (negative for alterations in *EGFR*, *KRAS*, *BRAF*, *MEK1*, *HER2* and *ALK*)

Unlike in ALK, where the fusion partner provides a dimerisation domain that induces constitutive oligomerisation and thus activation of the kinase, the mechanism by which ROS1 fusion proteins become constitutively active is not exactly known. In fact, many of the known ROS1 fusion partners do not contain dimerisation domains [22]. What is known is that several signalling pathways are activated by ROS1 fusion proteins. Expression of FIG-ROS1, CD74-ROS1 or SCD4-ROS1 in fibroblasts or Ba/F3 cells has been shown to result in auto-phosphorylation of ROS1 and phosphorylation of SHP-2, MAP-ERK kinase, ERK, STAT3 and AKT, and these effects have been blocked by pharmacological inhibition of ROS1. Subcellular localisation and downstream signalling may differ depending on the fusion partner of ROS1 [19, 20, 25, 26], but in general, the activated pathways seem to involve common growth and survival pathways that are also activated by other RTKs.

## Efficacy and safety of ROS1 inhibitor therapy

ROS1 inhibition by crizotinib has been studied in a number of early-phase clinical trials in patients with advanced *ROS1*-positive NSCLC (Table 2). In the *ROS1* expansion cohort of a phase 1 trial of crizotinib, the objective response rate (ORR) was 72 %. Median duration of response was 17.6 months and median progression-free survival (PFS) was 19.2 months. No relationship was observed between *ROS1* fusion partner and duration of crizotinib treatment [8]. Furthermore, ORR with crizotinib was 80 % and median PFS was 9.1 months in heavily pre-treated patients in a retrospective study [27]. Consistent with this, in patients with advanced *ROS1*-positive NSCLC receiving crizotinib in a French phase 2 trial, ORR was 69 % and median PFS was 9.1 months [28]. Finally, ORR was 69 % and median PFS was 12.9 months with crizotinib in a phase 2 trial in East Asian patients with advanced *ROS1*-positive NSCLC [29]. Across the clinical studies in *ROS1*-positive NSCLC, crizotinib treatment was well tolerated, with an adverse event profile similar to that seen in *ALK*-positive NSCLC [3, 30]. Phase 2 trials in *ROS1*-positive NSCLC are currently ongoing.

**Table 2:** Summary of the clinical studies of crizotinib in *ROS1*-positive NSCLC

Trial (clinicaltrials.gov I.D.)	Phase	Number of patients	Status	Outcomes
PROFILE 1001 (NCT00585195)	1	50	Data published [8]	ORR 72 % Median duration of response 17.6 months Median PFS 19.2 months 12-month OS 85 %
EUROS1	Retrospective study	32	Data published [27]	ORR 80 % Median PFS 9.1 months
AcSé (NCT02034981)	2	37	Data presented [28]	ORR 69 % Median PFS 9.1 months
OxOnc (NCT01945021)	2	127	Data presented [29]	ORR 69 % Median PFS 13.4 months
EUCROSS (NCT02183870)	2	30 (estimated)	Ongoing	N.A.
METROS (NCT02499614)	2	40 (estimated)	Ongoing	N.A.

*N.A.* not available, *NSCLC* non-small cell lung cancer, *ORR* objective response rate, *OS* overall survival, *PFS* progression-free survival

## Detection of *ROS1* gene rearrangements

As mentioned above, *ROS1* gene rearrangement is one of several addictive oncogenic events which may drive a proportion of pulmonary adenocarcinomas. Since *ROS1*-positive tumours are very sensitive to treatment with tyrosine kinase inhibitors such as crizotinib, detecting this rare genetic alteration may be an important step in the diagnostic work-up of a patient with lung adenocarcinoma.

The traditional approach to detecting *ROS1* gene rearrangement is by the use of so-called dual ‘break-apart’ fluorescence in situ hybridisation (FISH) probes, where the rearrangement separates the two ends of the *ROS1* gene and thus the two probes. The rearrangement event, when oncogenic, fuses the portion of the *ROS1* gene bearing the tyrosine kinase domain with another partner to create a *ROS1* fusion gene. An alternative approach to the identification of the abnormal DNA sequence created by the rearrangement event is to use massive parallel ‘next-generation’ sequencing (NGS). A variety of approaches using this technology may be used, and commercial platforms are now available, for use with test kits covering a range of fusion genes, including *ROS1*. Following transcription, fusion gene mRNA provides another possibility for detection with polymerase chain reaction (PCR) technology using a multiplex platform capable of detecting a range of known *ROS1* fusion gene transcripts. For oncogenic activity, the *ROS1* fusion gene transcript must be translated into protein with tyrosine kinase activity. Elevation of ROS1 protein levels in tumour cells may be detected by immunohistochemistry (IHC) using antibodies against ROS1 protein. This leaves open a possible scenario, rather like testing for *ALK* gene rearrangement, where elevations in the protein may be used as a surrogate marker for the presence of a *ROS1* gene rearrangement. Furthermore, a positive IHC test is likely to be an indication of a functional rearrangement, since the protein must be present for oncogenic activity and the protein tyrosine kinase is the target of drug therapy. Details of these various testing approaches are discussed below.

As with all current biomarker testing in non-small cell carcinomas, adequate quality and quantity of tissue is required for testing, and this has been extensively discussed elsewhere [31–34]. Issues relating to pre-analytics are discussed below. For many laboratories, *ROS1* testing is not yet a routine. Instead, it may be a test considered after several more routine tests, such as *EGFR* or *KRAS* mutation and *ALK* gene rearrangement, prove negative. Consequently, the samples to be used for *ROS1* testing may have been exhausted by prior tests, placing the test at risk when pursued in this way. As *ROS1* testing becomes more routine, driven by drug approval and availability, or when *ROS1* detection is more generally covered within a targeted NGS gene panel, these risks should diminish.

### Fluorescence in situ hybridisation

The screening strategy for *ROS1* rearrangement was developed based on the experience of *ALK* testing in lung adenocarcinomas [17, 23, 32]. While *ROS1* alterations may be detected with a variety of techniques, most laboratories rely on FISH assays using a dual-colour break-apart probe design. These involve labelling the 3′ (centromeric) part of the fusion breakpoint with one fluorochrome and the 5′ (telomeric) part with another fluorochrome. It is important to choose a 3′ probe colour [16, 22, 35–37] that allows *ROS1* (most commonly a green 3′ fluorochrome) and *ALK* (an orange 3′ fluorochrome) tests to be distinguished [38], particularly if the two tests are to be run together on one slide, in parallel.

**Fig. 2:**
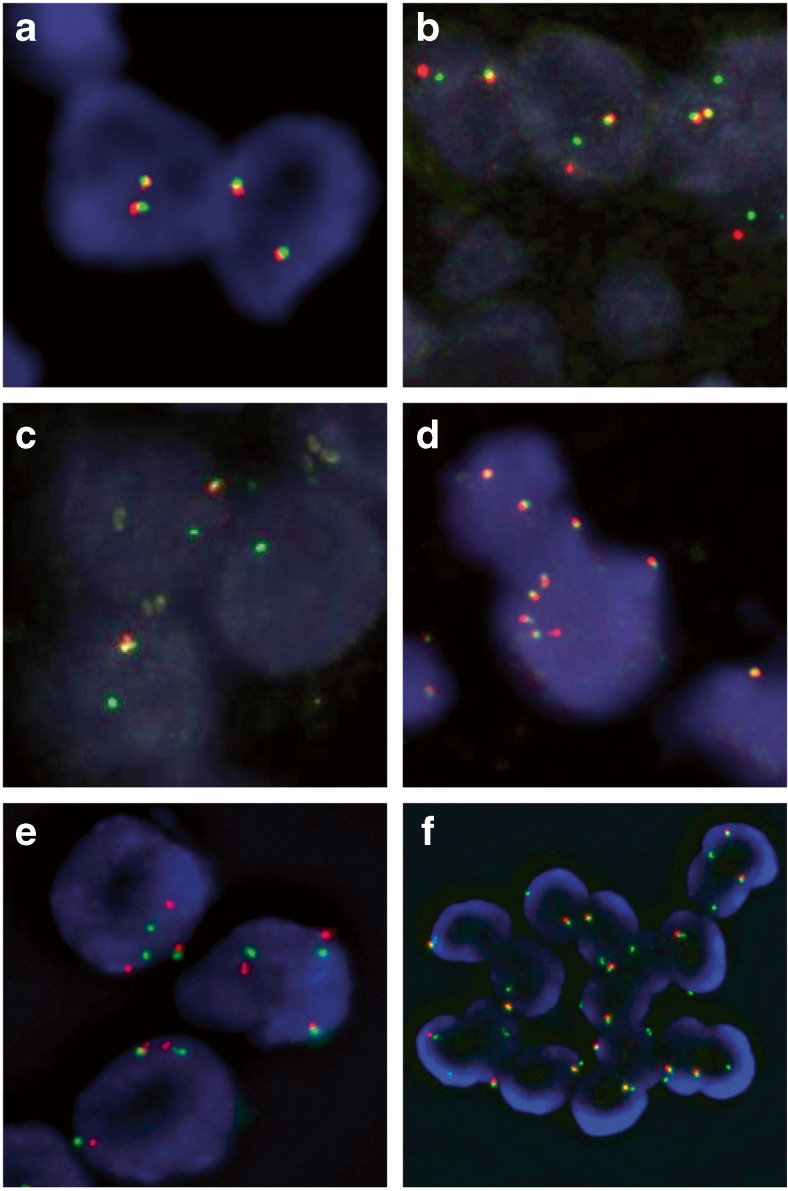
Examples of different FISH signal patterns using *ROS1* break-apart assays. **a–d** Vysis LSI ROS1 (Cen) SpectrumGreen Probe and Vysis LSI ROS1 (Tel) SpectrumOrange Probe (Abbott Molecular, IL, USA) on histological specimens. **a** Normal (negative) *ROS1* pattern: two fused signals. **b** Typical *ROS1*-positive pattern with fused and split signals. **c** Atypical *ROS1*-positive pattern with one fusion signal and isolated 3′ green signals. **d** Increased *ROS1* copy number. This pattern should not be interpreted as positive; **e–f** ZytoLight SPEC ROS1 (Cen) Green Probe and (Tel) Orange Probe (ZytoVision, Bremerhaven, Germany) on cytological specimens. **e** Split signals. **f** Isolated 3′ green signals

There are two positive *ROS1* rearrangement patterns. One is the break-apart pattern (‘classic’ pattern) with one fusion signal (native *ROS1*) and two separated 3′ and 5′ signals. The other positive pattern is an isolated 3′ signal pattern, usually an isolated green signal, (‘atypical’ pattern) with one fusion signal (native *ROS1*) and one 3′ signal without the corresponding 5′ signal (Fig. 2). Table 3 summarises the criteria for *ROS1* FISH interpretation in NSCLC [23, 32, 35, 36].

**Table 3:** Criteria for dual-colour break-apart FISH detection of *ROS1* rearrangements in NSCLC

	Positivity criteria
Number of cells counted	At least 50 tumour cells (first step); 100 cells (second step)
Patterns for positivity	Typical pattern: two separated 3′ and 5′ plus one fusion signal; Atypical pattern: isolated 3′ signal plus one fusion signal
First step	
Score of positivity	25 positive cells out of 50 tumour cells
Negativity	Less than 5 positive tumour cells
Equivocal	5–25 positive cells (need second observer for an additional cell count reading)
Second step (for an equivocal result)	
Positivity threshold (additional cell count reading)	≥15 % positive cells out of 100 tumour cells
Gene copy number alterations	Not rearranged

*NSCLC* non-small cell lung cancer

For optimal FISH results, there are a number of relevant factors. Use of sections older than 6 months may result in poor hybridisation. In the post-analytic phase, it is important that only intact tumour cells with non-overlapping nuclei are scored. Furthermore, the use of an automated software system (e.g. BioView Duet system, Rehovot, Israel) can facilitate FISH scoring. It should be noted that FISH testing for *ROS1* (and *ALK*) is not restricted to histological tissue sections, but is also applicable to cytological specimens [39, 40].

### Immunohistochemistry

**Fig. 3:**
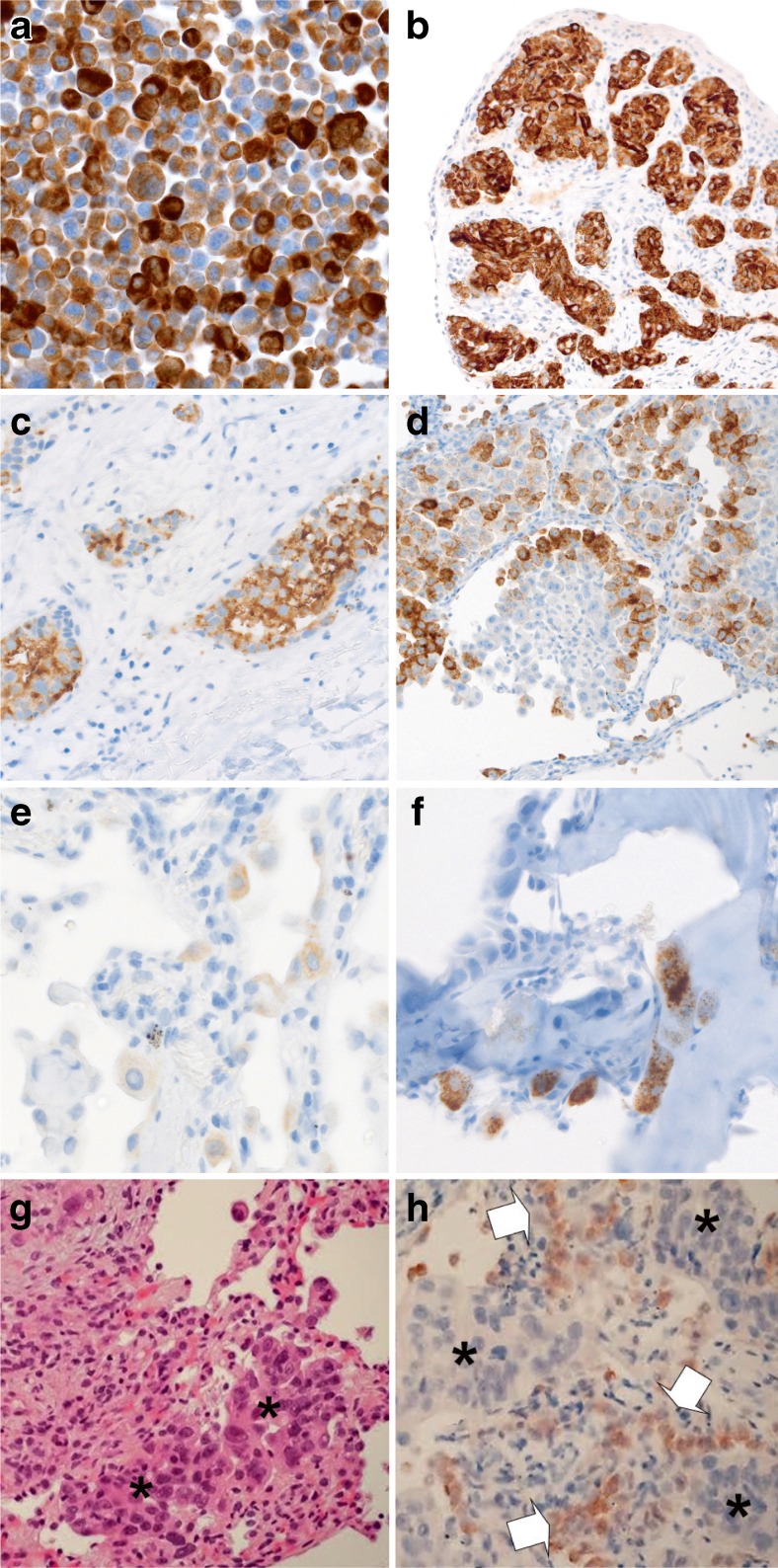
**a**–**f** Examples of ROS1 IHC in histological NSCLC specimens (D4D6 antibody, Ventana BenchMark XT; DAB chromogen). **a** HCC78 cell line (cellblock; ×400). **b** NSCLC with diffuse, strongly positive staining (×200). **c** NSCLC with diffuse, granular cytoplasmic staining (×400). **d** Adenocarcinoma with heterogeneous staining (×200). **e** Non-neoplastic type II pneumocytes with weak ROS1 staining (×630). **f** Bone metastasis of a *ROS1*-negative NSCLC showing strong granular staining of non-neoplastic osteoclastic giant cells (×400). **g–h** Aberrant immunostaining of ROS1 in a transbronchial biopsy with lung adenocarcinoma. **g** H&E stain, *asterisks* show tumour cells. **h** ROS1 IHC in adjacent hyperplastic type II pneumocytes (*arrows*) but not in tumour cells (*asterisks*)

**Fig. 4:**
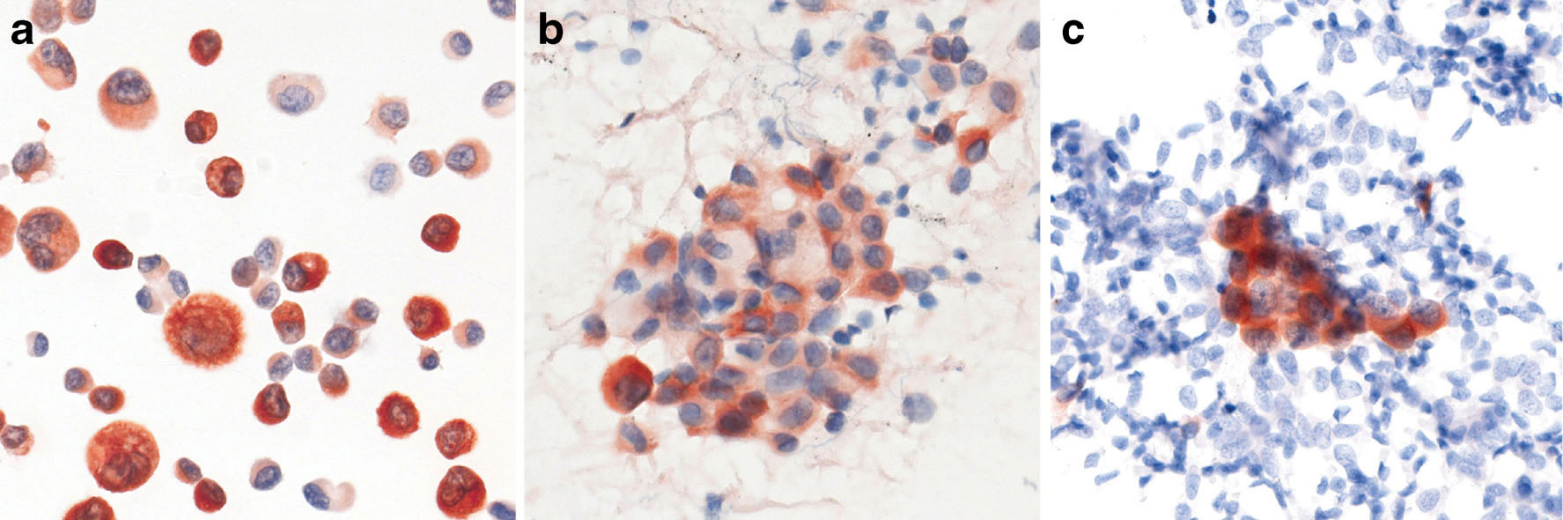
ROS1 IHC in ethanol-fixed and previously Papanicolaou-stained cytological specimens (D4D6 antibody, Leica BondMax; AEC chromogen, ×400). **a** HCC78 cell line (positive control; cytospin). **b** *ROS1*-positive adenocarcinoma. **c** Small group of *ROS1*-positive adenocarcinoma cells surrounded by numerous benign respiratory epithelial cells

Given the rarity of *ROS1* rearrangements in NSCLC, screening of tumours by IHC may allow unnecessary FISH analysis in *ROS1*-negative cases to be avoided and thus dramatically reduce the cost of testing. Based on available data, IHC is an effective screening tool to detect *ROS1*-positive NSCLC, with a sensitivity of 100 % in most studies and a variable specificity ranging from 92 to 100 %, depending on the threshold used to define positivity [20, 36, 37, 41–45]. These results are based on the use of the ROS1 (D4D6) rabbit monoclonal antibody (Cell Signaling Technology, Danvers, MA, USA) applied at dilutions ranging from 1:50 to 1:1000 with various antigen retrieval methods and use of different amplification and detection systems, in automated instruments or manually. Tumour specimens with known *ROS1* rearrangement, or a cellblock of the HCC78 cell line harbouring the *SLC34A2-ROS1* fusion gene, can serve as positive controls [20] (Figs. 3a and 4a). In contrast to ALK, where the ganglion cells of the appendix serve as an adequate external control, there is currently no good external benign tissue control for ROS1.

Currently, there is no universally accepted system for how to score IHC results. The thresholds used include either any staining above faint background (if present) or moderate or strong staining (2+/3+). Another option is the use of an H-score with optimal threshold for ROS1 positivity defined as >100 [40] or >150 [43]. Notably, weak and focal staining was found in 31 % of 253 *ROS1* wild-type lung carcinomas in one study [44]. However, this had hardly any influence on specificity when appropriate thresholds of positivity were used (i.e. H-score >150). Thus, none of the reported scoring methods have been shown to be clearly superior to the others since all resulted in very good to excellent correlation with FISH results.

Positive ROS1 IHC typically reveals finely granular cytoplasmic staining (Fig. 3). However, the staining pattern may depend on the function and subcellular location of the gene fusion partner [44, 46]. Globular ROS1 immunoreactivity has been described in tumour specimens with the *CD74*-*ROS1* fusion, and membranous staining has been observed in tumours with the *EZR-ROS1* fusion [41, 44]. Interestingly, ROS1 expression levels in *ROS1*-positive lung cancers and cell lines can vary from cell to cell, suggesting dynamic ROS1 protein expression despite homogeneous presence of *ROS1* gene rearrangement (Figs. 3 and 4). Detection of ROS1 protein expression in *ROS1*-positive adenocarcinomas with signet ring cells is challenging since the cytoplasm is largely replaced by non-reactive mucin [44]. The same pitfall has already been shown for ALK IHC [47]. One should be aware that weak ROS1 expression is occasionally detectable in non-neoplastic hyperplastic type II pneumocytes (Fig. 3e, h) and in alveolar macrophages. In bone metastases, there is strong granular cytoplasmic staining of osteoclast-type giant cells (Fig. 3f).

Lung cancer is often diagnosed by cytology alone, necessitating *ROS1* testing in cytological specimens. For cellblocks, the same IHC protocols can be used as for formalin-fixed paraffin-embedded (FFPE) tissue specimens. In the laboratory of one of the authors (L.B.), ROS1 IHC on the BondMax immunostainer is routinely used on conventional, ethanol-fixed and Papanicolaou-stained cytological specimens, including smears or cytospin preparations (Fig. 4). Notably, in the case of limited cytological material, FISH for confirmation of a positive IHC result can be applied to the immunostained slide if 3-amino-9-ethylcarbazole (AEC) was used as a red chromogen. Although IHC on cytological specimens is common practice in many laboratories, immunocytochemistry performed on smears and/or cytospin slides may be much more influenced by various pre-analytical factors [48, 50]. Thus, it should be performed only in laboratories with experience and appropriate quality assurance in place. The simultaneous use of cell blocks would allow testing sequentially for several biomarkers.

ROS1 IHC has a great advantage over FISH in that it can detect rare positive cells or cell groups within a majority of non-neoplastic reactive cells that would be easily missed by FISH. This can be particularly helpful in cytological specimens where architectural tissue context is lost. Although it has been proposed to score ROS IHC only in specimens containing ≥20 tumour cells [41], a positive result in even only a few clearly neoplastic cells can be considered diagnostic.

In summary, IHC is a cost-effective method that can be used to efficiently screen patients with lung cancer for *ROS1* rearrangements. Given the laboratory-dependent variability of specificity, confirmation of positive or doubtful ROS1 IHC by FISH or another method is highly recommended.

### Non-in situ technologies

In addition to FISH and IHC, a number of non-in situ approaches based on real-time PCR (RT-PCR) or NGS have been developed for the detection of *ROS1* gene rearrangements. RT-PCR assays require multiple specific primer sets to discriminate amongst known fusion variants, which can be confirmed by subsequent sequencing [50]. The breakpoints of *ROS1* are located at exons 32, 34, 35 and 36, and the most frequent ROS1 fusion partners include *SLC34A2*, *CD74*, *TPM3*, *SDC4*, *EZR*, *LRIG3*, *FIG* or *GOPC*, *MSN*, *KDELR2* and *CCDC6* [18, 19, 21, 22, 51]. RT-PCR has been successfully utilised to identify positive cases with a sensitivity of 100 % and a specificity of 85–100 %, using FISH as the reference standard method [37, 42]. Multiplex RT-PCR is easy to perform, rapid and relatively inexpensive but may be challenging using RNA extracted from FFPE samples [52]. In addition, as the list of *ROS1* fusion partners is quite large and still growing, RT-PCR is likely to miss rare variants. These reasons have limited the use of the technique in clinical practice. Recently, a very sensitive RT-PCR-based method was devised to detect the overexpression of 3′ regions of fusion transcripts involving tumour genes constitutionally repressed or expressed at very low levels [53]; this approach has been successfully applied to *ALK* gene fusions in lung cancer [53, 54]. Unfortunately, this method cannot be easily applied to *ROS1*, since the gene is also expressed in normal and hyperplastic lung tissue [15, 55]. An alternative transcript-based method for detecting *ROS1* fusion genes is also available. The NanoString assay, capable of detecting known fusion gene transcripts and employing a dual capture and reporter probe system, provides a convenient and commercially available assay that has shown good concordance with FISH and IHC results for ROS1 [50, 55].

A series of innovative approaches to detect gene fusions in multiple targets has been developed using NGS (Table 4). It is remarkable that some of these comprehensive assays require as little as 10 ng of RNA [56], with relatively low failure rates in paraffin-embedded tissue (5.6 % in the authors’ experience [unpublished data]). A very sensitive NGS technique to assess *ROS1* and other gene rearrangements in lung cancer is anchored multiplex PCR that targets only the gene of interest, allowing the detection of the specific alteration irrespective of fusion partner. Validation of a gene rearrangement panel using 319 FFPE samples showed 100 % sensitivity and 100 % specificity compared with reference assays [51].

**Table 4:** NGS strategies for the detection of gene fusions

Enrichment method for NGS	Reference
Hybrid capture-based target enrichment	Drilon et al. [86]
Multiplex amplicon RNA massive parallel sequencing	Moskalev et al. [54]
Personalised analysis of rearranged ends (PARE)	Leary et al. [87]
Anchored multiplex PCR (AMP)	Zheng et al. [51]

*NGS* next-generation sequencing

These promising results suggest potential application of non-in situ methodologies in clinical practice, as stand-alone methods or as complementary tests within algorithms for the selection of patients to be treated with *ROS1*, *RET* or *NTRK* inhibitors [57]. However, published data for these assays are still limited.

### Concordance between FISH, IHC and PCR

There is good correlation between FISH and IHC using clone D4D6 with a highly sensitive amplification kit. Although some discrepant cases have been reported, ROS1 testing by IHC seems to be highly sensitive, but less specific, also when compared with ALK IHC for detection of the corresponding gene rearrangement. As suggested by others [41], IHC testing of specimens containing at least 20 tumour cells and application of an H-score cut-off of >100 are highly concordant with *ROS1* rearrangement by FISH or RT-PCR.

Currently, there is very limited published information on the concordance of IHC, in situ hybridisation (ISH) and non-in situ tests for the detection of *ROS1* gene rearrangements in lung adenocarcinoma [36, 37, 42, 58]; less than 30 cases with gene rearrangements have been subjected to comparative study of the three methods. Four of the *ROS1* tests currently hold in vitro diagnostic (IVD) and CE-marked status (Table 5). The general consensus seems to be that IHC, ISH and non-in situ methods all are promising for the detection of *ROS1*-positive cases, with concordance rates well above 90 %. Preliminary conclusions from limited studies suggest a role for IHC as a screening tool, but so far the lack of an IVD-classified IHC assay is problematic. Advantages of RT-PCR analysis include the highest sensitivity reported and the ability to identify translocation partners. On the other hand, there are potential issues around the quality and quantity of mRNA that may be obtained from routine, FFPE NSCLC diagnostic tissues. Furthermore, familiarity and availability of FISH as a technique in detecting other markers, such as ALK, is also important. Perhaps the use of more than one technique could be of value until further experience of testing and companion diagnostics with IVD status have emerged.

**Table 5:** Commercially available assays for *ROS1* testing

Method	Manufacturer	Reagent	Regulatory status
FISH	Cytocell	ROS1 Dual Color Break Apart Probe	CE-IVD
	ZytoVision/Zytomed	ZytoLight SPEC ROS1 Dual Color Break Apart Probe	CE-IVD
	Abbott	ROS 1 Break-Apart FISH	RUO
IHC	Cell Signaling Technologies	ROS1 D4D6 rabbit monoclonal antibody	RUO
RT-PCR	AmoyDx	ALK and ROS1 gene fusion detection kit	CE-IVD
NGS	Thermo Fisher	Oncomine Fusion panel (ALK, ROS1, RET and NTRK1)	CE-IVD
	ArcherDx	FusionPlex™ ALK, RET, ROS1 v2 Panel	RUO

*FISH* fluorescence in situ hybridisation, *IHC* immunohistochemistry, *IVD* in vitro diagnostic, *NGS* next-generation sequencing, *RT-PCR* reverse transcription polymerase chain reaction, *RUO* research use only

## General recommendations for *ROS1* testing

Current guidelines either do not refer to *ROS1* testing [59] or mention it briefly without making any strong recommendation [33]. With recent changes in the status of crizotinib for the treatment of *ROS1*-positive NSCLC, the case for recommending *ROS1* testing will now increase. Certainly, assuming drug treatment is available, response rates in treated patients whose tumours bear a *ROS1* rearrangement are impressive [8].

The group of patients more likely to bear a *ROS1* fusion gene is largely the same as that currently recommended for testing for *EGFR* gene mutation and *ALK* gene rearrangement. Clinical features such as gender, ethnicity and smoking status are not used to select patients for *EGFR* or *ALK* testing [33, 59].

Currently, however, as mentioned above, *ROS1* testing is often part of a second phase of testing in a patient whose tumour is negative for more common, routinely tested alterations such as *EGFR* and *KRAS* mutation and *ALK* gene rearrangement and who is a lifelong never or long-time ex-smoker. This is based upon the observations that these various addictive oncogenic driver events tend to be mutually exclusive in occurrence and are almost exclusively found in adenocarcinoma. As mentioned above, as NGS techniques are introduced into routine diagnostic practice, *ROS1* fusion gene testing will be provided as part of the testing ‘package’, whether or not this particular test was actively sought by a treating physician.

At present, when *ROS1* testing is required, it will be reasonable to test the same tumours currently being selected for *EGFR* mutation and *ALK* gene rearrangement. Although this should ideally occur in parallel, this is not possible in all cases. Therefore, in order to save tissue and time, it is wise to cut extra blank sections at the first cutting session [60]. FISH remains the core test for the time being. Laboratories may use IHC as a screening tool, but with our current state of knowledge, a positive IHC test should be confirmed by FISH testing. If laboratories chose to use a multiplex PCR approach instead of FISH, they should be aware of the possible pitfalls of this highly sensitive and specific technique, in terms of sample quality and risks of test failure.

There are a number of general principles that should be observed in order to ensure high-quality laboratory testing for predictive biomarkers in NSCLC. The use of adequate control materials, awareness that test outcomes may be confounded by pre-analytical issues in tissue handling and processing and the need for laboratories to participate in external quality assessment programmes are discussed in the following sections.

### Guidance on the use of controls

With ISH techniques, the case for study serves as a control when consistently presenting signals, both in tumour cells and in the accompanying normal cells (lymphocytes, fibroblasts, non-neoplastic lung epithelium). A pre-hybridisation assessment of digestion is useful in difficult samples (e.g. very small biopsies with low tumour content).

With IHC, ROS1 protein expression may also be detected in normal cells, namely histiocytes/giant cells, reactive type II pneumocyte hyperplasia and bronchiolar metaplasia at the tumour periphery or in subpleural areas. In most cases, the expression in these cells is weak to moderate (1+/2+ in intensity), and it is unclear whether protein stability may be affected by pre-analytical variables (e.g. time of fixation) [43]. To control for appropriate analytical conditions of ROS1 testing by IHC, it is mandatory to include a piece of tissue from a *ROS1* FISH-positive tumour on the same slide of the neoplasm of interest or on a separate slide to use in the same run.

### Pre-analytical variables and factors affecting quality of biopsies and surgical samples

Regarding the ROS1 epitope, the influence of pre-analytical factors has not been investigated systematically, but experiences from our group and several others [36, 61] show that the protein is relatively stable and may be detected reliably by IHC. In addition, the corresponding genomic alterations can be reproducibly detected by ISH [62]. To improve nucleic acid stability, new techniques of fixation may become useful [63]. Nonetheless, attention should be paid to a number of basic requirements in order to avoid false-negative results.

#### Resection material

Surgical material such as lobectomies should initially be handled macroscopically (documentation and gross sectioning) following a standardised protocol. To greater standardise the work-up of resection material, vacuum preservation might be considered [64]. It is important that these procedures are conducted in a standardised way.

#### Biopsies

Biopsies almost always are transferred into the fixation solution immediately after removal from the patient. The small tissue fragments should be fixed for no longer than 24 h; as shown for several antigens, a gradual decrease in antigenicity may appear over time. Prior or in parallel to *ROS1*, several immunohistochemical, ISH and molecular markers may need to be analysed to confirm the subtype of NSCLC and the immunological and molecular profile. This means that a minimum amount of tissue/cells is required for reliable analysis. In the context of personalised medicine, this multi-parameter analysis plays an increasing role, which has led to the suggestion to provide at least three to four endobronchial biopsy specimens for pathology.

### External quality assessment

For the successful treatment of patients, it is of great importance that molecular test results are accurate, highly reliable, clearly understandable to the clinician and reported within an acceptable turnaround time [59]. In 2012, the European Society of Pathology (ESP) proposed an external quality assessment (EQA) scheme to promote high-quality biomarker testing in NSCLC for *EGFR* mutation analysis and *ALK* rearrangement detection. From 2014 onwards, *ROS1* testing was also included [65]. The EQA was performed at the beginning of the development of *ROS1* testing. The rate of false negativity for IHC on a limited number of *ROS1*-positive cases was approximately 15 %. The rate of false positivity was <10 %. For FISH, although the number of evaluable cases was limited, no false-negative scores were present in a *ROS1*-positive control cell line. Overall, at an early stage of *ROS1* testing, the EQA showed promising results, emphasising the need for regular EQA monitoring.

## Integration of ROS1 into current testing algorithms

Since clinical trials with crizotinib in *ROS1*-positive patients have used FISH, this method has been considered the ‘gold standard’ for determining *ROS1* positivity by the FDA in the USA. European guidelines currently recommend *ROS1* rearrangement testing in patients with advanced NSCLC who have previously tested negative for *EGFR* mutation and *ALK* rearrangement, including all stage IIIB/IV histological subtypes in non-smokers and the non-squamous cell carcinoma subtype in current or ex-smokers [33]. As the demand for *ROS1* testing increases, it is reasonable that *ROS1* rearrangement be considered for testing concurrently with *ALK* rearrangement and *EGFR* mutation. Cutting extra blank sections for *ROS1* testing (and also for additional tests such as PD-L1) at the first cutting session is good practice to avoid tissue waste, especially when the amount of tumour tissue is scarce.

**Fig. 5:**
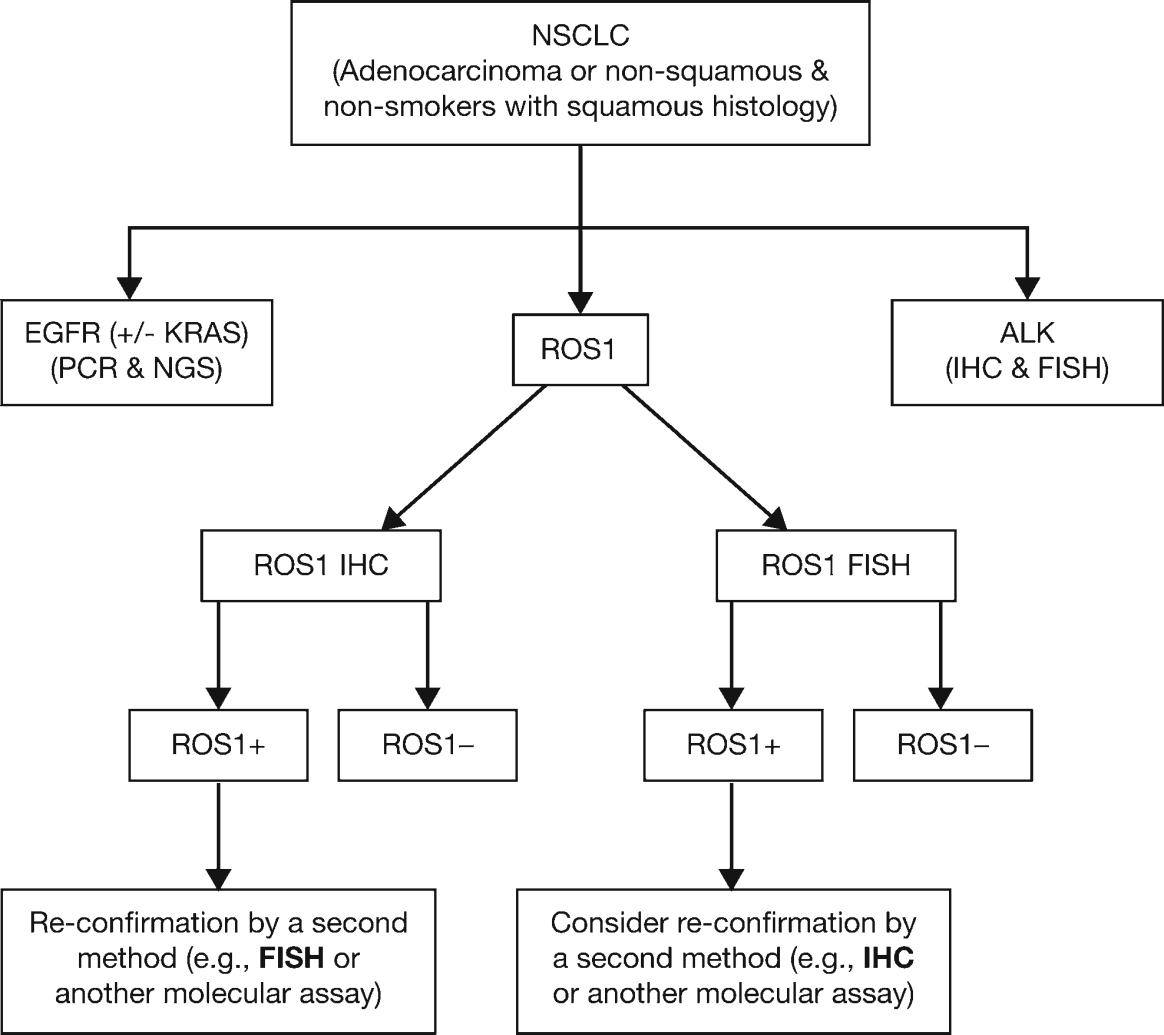
Algorithm for predictive genetic testing in advanced NSCLC: routine practice

Although validation with large series is needed, IHC could also become a good preliminary, rapid screening method. An algorithm based on IHC screening with further confirmation by a *ROS1* break-apart FISH assay in positive or doubtful cases seems appropriate. Nevertheless, in the near future the possibility of using transcript-based methods in a single-tube assay to detect several oncogenic fusions involving the *ALK*, *RET*, *ROS1* and *NTRK1* genes could drastically limit the use of IHC and FISH tests. The algorithm presented in Fig. 5 is proposed for use in routine clinical practice.

## Conclusions

With the recent FDA approval of crizotinib for patients with advanced *ROS1*-positive NSCLC, ROS1 fusion proteins comprise one of only three oncogenic drivers in NSCLC for which an approved targeted therapy is available. Like *ALK*, *ROS1* gene rearrangements may be detected using a break-apart FISH assay, IHC and a number of non-in situ methods. Although FISH was the methodology used in the clinical trials of crizotinib, IHC can also be used as a screening approach if carefully validated. The real-world possibility of false-positive FISH or IHC results strengthens the case for confirmation of positive cases by a second methodology. Targeted NGS is a valid alternative if cost and turnaround time are reasonable. As testing for *ROS1* becomes increasingly important for patients with advanced NSCLC, it will be key to share experience and recommendations on how to accurately implement these diagnostic methodologies into routine practice. Regardless of which testing method(s) is used, it is key that routine testing for *ROS1* in the clinical setting be carefully validated, with appropriate controls and participation in EQA schemes. To achieve efficient molecular testing in NSCLC and an optimal turnaround time for test results, we propose that *EGFR*, *ALK* and *ROS1* are tested for upfront and in parallel in NSCLC specimens.
